# Octyl Gallate Exhibits Trypanocidal Activity Through Trypanothione Reductase Inhibition and Immunomodulation In Vitro

**DOI:** 10.3390/biomedicines14071471

**Published:** 2026-06-29

**Authors:** Vanessa Maria Rodrigues de Souza, Airton Lucas Sousa dos Santos, Yasmim Alves Aires Machado, Franciregina Silva Araújo, Julyanne Maria Saraiva de Sousa, Raiza Raianne Luz Rodrigues, José Wheslley Rodrigues de Lucena, Sônia Nair Báo, Ingrid Gracielle Martins da Silva, Karine Brenda Barros-Cordeiro, Paulo Sérgio de Araujo Sousa, Jefferson Almeida Rocha, Leiz Maria Costa Véras, Thaís Amanda de Lima Nunes, Marcos Vinícius da Silva, Klinger Antonio da Franca Rodrigues

**Affiliations:** 1 Infectious Disease Laboratory, Campus Ministro Reis Velloso, Federal University of Parnaíba Delta, Parnaíba 64202-020, PI, Brazil; rodriguesvanessa745@gmail.com (V.M.R.d.S.); sousaairtonlucas@gmail.com (A.L.S.d.S.); wheslleyrodriguess@ufdpar.edu.br (J.W.R.d.L.); 2 Microscopy and Microanalysis Laboratory, Department of Cell Biology, Institute of Biological Sciences, University of Brasília, Brasília 70910-900, DF, Brazil; snbao@unb.br (S.N.B.); gracilias@gmail.com (I.G.M.d.S.); karine.brenda22@gmail.com (K.B.B.-C.); 3 Research Group in Medicinal Chemistry and Biotechnology, Federal University of Maranhão ( UFMA), São Bernardo 65550-000, MA, Brazil; psergio.araujosousa@gmail.com (P.S.d.A.S.); jeffersonbiotec@gmail.com (J.A.R.); 4 Graduate Program in Biotechnology, Parnaíba Delta Federal University, 2819, São Sebastião Avenue, Parnaíba 64202-020, PI, Brazil; leiz.veras@gmail.com; 5 Laboratory of Immunology and Parasitology, Institute of Biological and Natural Sciences, Federal University of Triângulo Mineiro, Uberaba 38025-180, MG, Brazil; thaisaln13@gmail.com (T.A.d.L.N.); marcos.silva@uftm.edu.br (M.V.d.S.); 6 Center for Basic and Applied Immunology, Dom Delgado University City, Federal University of Maranhão, São Luiz 65065-545, MA, Brazil

**Keywords:** antioxidant, chagas disease, macrophages, neglected diseases

## Abstract

**Background/Objectives:** American trypanosomiasis, caused by *Trypanosoma cruzi*, remains a major public health challenge due to the limited efficacy and adverse effects associated with current treatments. Octyl gallate (OG), a semi-synthetic derivative of gallic acid, has demonstrated promising biological activities, including antiparasitic effects. **Methods:** The in vitro trypanocidal activity of OG was evaluated against *T. cruzi*. Mechanism of action studies included the inhibition of the trypanothione reductase enzyme and flow cytometry assays to measure cell death pathways (propidium iodide uptake). Additionally, the immunomodulatory potential of the compound was investigated by assessing cytokine production and innate immune responses. **Results:** In this study, the trypanocidal activity of OG against different evolutionary forms of *T. cruzi* was investigated. Using MTT-based viability assays, OG exhibited significant activity against epimastigotes (IC50 = 5.92 ± 0.47 µM), trypomastigotes (EC50 = 3.20 ± 0.14 µM), and intracellular amastigotes (EC50 = 4.07 ± 0.72 µM). The compound also demonstrated favorable selectivity indices, particularly against trypomastigotes and amastigotes, indicating selective toxicity toward the parasite compared to mammalian host cells. In infected macrophages, OG increased TNF-α and IL-12 production while reducing IL-10 and IL-6 levels, in addition to stimulating reactive oxygen species (ROS) and nitric oxide (NO) production, suggesting an immunomodulatory effect that contributes to parasite control. Molecular docking analyses revealed a favorable interaction between OG and trypanothione reductase (TR), while biochemical assays demonstrated reduced NADPH consumption, indicating interference with TR activity. Ultrastructural analysis revealed severe morphological alterations, including membrane disruption, cytoplasmic disorganization, mitochondrial swelling, and features consistent with apoptosis-like cell death. **Conclusions:** Collectively, these findings demonstrate that OG exhibits potent and selective trypanocidal activity associated with immunomodulatory effects, ultrastructural damage, and disruption of parasite redox metabolism through TR inhibition, supporting its potential as a candidate for future preclinical studies against Chagas disease.

## 1. Introduction

Chagas disease (CD), also known as American trypanosomiasis, is caused by the hemoflagellate protozoan *Trypanosoma cruzi*. According to the World Health Organization (WHO), approximately 6–7 million people are infected worldwide, predominantly in Latin America, although cases have increasingly been reported in North America, Europe, and Western Pacific countries [1].

The disease is transmitted mainly through contact with feces and urine of infected triatomine vectors. However, oral transmission through contaminated food and beverages has emerged as a major epidemiological concern in recent years, particularly in Latin America, where several outbreaks have been associated with the consumption of contaminated açaí, sugarcane juice, palm hearts, and other food products [2].

Clinically, CD presents two distinct phases: acute and chronic. During the acute phase, parasites replicate and disseminate through the bloodstream. If untreated, the infection progresses to the chronic phase, characterized by parasite persistence in cardiac and gastrointestinal tissues, leading to chronic inflammation and severe complications, including chagasic cardiomyopathy and digestive disorders [3].

Despite more than a century of scientific advances, pharmacological treatment of CD remains limited. Currently, benznidazole and nifurtimox are the only drugs available for therapeutic use. Although effective during the acute phase, both drugs show limited efficacy in chronic infections and are associated with significant adverse effects, including neurological, gastrointestinal, and dermatological disorders, frequently leading to treatment discontinuation [4].

Throughout history, humans have relied on natural resources to meet basic needs, including food and disease treatment [5]. Plants represent an important source of bioactive compounds, particularly antioxidant molecules, which have attracted considerable interest due to their ability to neutralize oxidative stress and protect cells from damage associated with several pathological conditions [6]. Antioxidant compounds have been extensively investigated and have demonstrated beneficial effects against cardiovascular, metabolic, gastrointestinal, microbial, and parasitic diseases, including Chagas disease [7].

Among plant-derived antioxidant compounds, phenolic acids and their derivatives have received particular attention because of their diverse biological activities. One example is octyl gallate (OG), a semi-synthetic derivative of gallic acid with demonstrated antioxidant, anti-inflammatory, neuroprotective, antibacterial, and antiparasitic properties [8,9,10] OG can be obtained from various plant sources, including *Camellia sinensis* (L.) *Kuntze*, *Vaccinium myrtillus* L., *Rubus idaeus* L., and *Persea americana* Mill [11]. Previous studies have demonstrated its activity against bacteria, fungi, protozoa, and viruses, including SARS-CoV-2 [12,13]. However, information regarding its activity against *T. cruzi* remains scarce. Therefore, the present study aimed to evaluate the trypanocidal activity of OG against different evolutionary forms of *T. cruzi* and its potential mechanisms of action.

## 2. Material and Methods

### 2.1. Reagents and Drugs

Liver Infusion Tryptose (LIT-NaCl 4 mg/mL; Na_2_HPO_4_. 12H_2_O 11.6 mg/mL; KCl 0.4 mg/mL; glucose 2.2 mg/mL; tryptose 5 mg/mL; liver infusion 5 mg/mL; hemin 25 mg/mL), Dulbecco’s modified Eagle’s medium (DMEM), 3-(4,5-dimethylthiazol-2yl)-2,5-diphenyltetrazoline bromide (MTT), stabilized antibiotic solution (penicillin 10,000 IU/mL; streptomycin 10 mg/mL), stabilized antibiotic–antimycotic solution (penicillin 10,000 IU/mL; streptomycin 10 mg/mL; amphotericin B 25 mg/mL), sodium cacodylate, lead citrate, and Trypan blue were purchased from Sigma-Aldrich (St. Louis, MO, USA). The drug benznidazole was purchased from the Pharmaceutical Laboratory of the State of Pernambuco (LAFEPE, PE, Brazil). Fetal bovine serum (FBS) was purchased from Cultilab (São Paulo, SP, Brazil). DMSO–Dimethyl sulfoxide and sodium dodecyl sulfate (SDS) were obtained from Mallinckrodt Chemicals (St. Louis, MO, USA). Glutaraldehyde, osmium tetroxide, uranyl acetate and Spurr resin were purchased from Electron Microscopy Sciences, (Hatfield, PA, USA). An FITC-Annexin V apoptosis detection kit with PI was purchased from Biolegend (San Diego, CA, USA).

**Figure 1:**
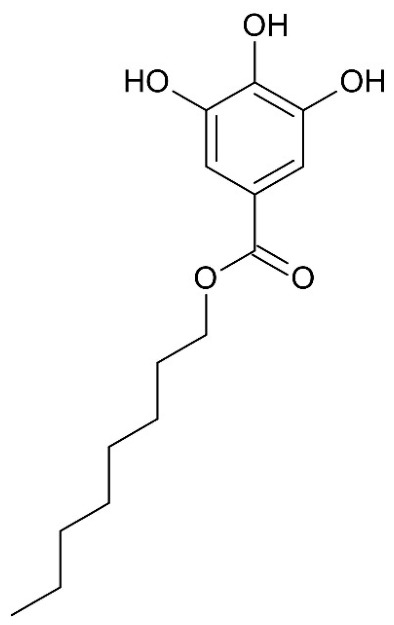
Chemical structure of octyl gallate (OG).

OG (Figure 1) was commercially purchased from Merck Life Science (St. Louis, MO, USA), with 99% purity. The OG compound was solubilized in DMSO–Dimethyl sulfoxide. Stock solutions were diluted to 20 mg/mL, not exceeding a final concentration of 0.5% DMSO.

### 2.2. Cultivation of Parasites

*T. cruzi* epimastigote forms, (strain CL) were cultured in LIT medium, pH 6.9, supplemented with 10% FBS, 1% penicillin (10,000 IU/mL), and streptomycin (10 mg/mL) (hereafter referred to as complete LIT) and maintained under BOD (Biochemical Oxygen Demand) incubator SolidSteel (São Paulo, SP, Brazil) at 28 °C.

Trypomastigote forms were obtained in vitro through infection of the LLC-MK2-CCL-7 cell line (10^7^ parasites/cell) grown in 75 cm^2^ cell culture flasks with DMEM supplemented. The cultures were incubated at 37 °C with 5% CO_2_. Every 48 h, the supernatant from the flasks containing the trypomastigote forms was centrifuged and the cells were washed twice with PBS and new medium was added. The resulting pellet, rich in trypomastigotes, was used directly in the biological assays [14].

### 2.3. Cultivation of Cell Lines

LLC-MK2 CCL-7, Vero CCL-81, and RAW 264.7 macrophage cells were purchased from the Rio de Janeiro Cell Bank and cultured in 75 cm^2^ cell culture flasks using DMEM supplemented with 10% FBS, 1% penicillin (10,000 IU/mL), and streptomycin (10 mg/mL) (hereafter referred to as complete DMEM), and incubated at 37 °C with 5% CO_2_. Subculturing was performed when the cells reached 80% confluence. During this process, the flasks were washed twice with phosphate-buffered saline (PBS), and fresh complete DMEM was added. Adherent cells were trypsinized and then centrifuged at 112× *g* for 10 min, stained with Trypan blue and counted in a Neubauer Chamber to ensure adequate distribution, at a concentration of 5 × 10^4^ cells/flask [14].

### 2.4. Anti-Trypanosoma Activity of OG on Evolutionary Forms of T. cruzi

#### 2.4.1. Anti-Trypanosoma Activity on Epimastigote and Trypomastigote Forms

To evaluate the anti-epimastigote and anti-trypomastigote activity, OG was added in serial concentrations (3.12 μM, 6.25 μM, 12.5 μM, 25 μM, 50 μM, and 100 μM) in 96-well culture plates containing complete LIT medium. Epimastigote and/or trypomastigote forms in the logarithmic growth phase (1 × 10^7^ per well) were then added. The plate was incubated at 28 °C in a BOD incubator for 72 h. To evaluate viability after treatment, 10 μL of MTT was added to each well, followed by incubation for an additional 4 h. Subsequently, 50 μL of a sodium dodecyl sulfate (SDS) solution was added to solubilize the formazan crystals, and the plate was again incubated overnight. Absorbance was measured using a spectrophotometer at 540 nm (model Molecular Devices Spectramax 190) (Curitiba, PR, Brazil). The results were expressed as percentage growth inhibition and IC_50_ values for the epimastigote forms. The control group, treated with complete LIT medium containing 0.5% DMSO, was considered as 0% parasite growth inhibition or viability. As a positive control, benznidazole was used at concentrations of 3.12–100 µM [14].

#### 2.4.2. Anti-Trypanosoma Activity on Intramacrophagic Amastigote Forms

Sterile round glass coverslips (13 mm) were placed in a 24-well plate, and RAW 264.7 macrophages were added to each well at a ratio of 1 × 10^5^ cells/mL in complete DMEM. The plate was incubated at 37 °C in 5% CO_2_ for 4 h. After this, the wells were washed and 1 mL of DMEM containing 1 × 10^6^ trypomastigotes (10:1 parasite:host cell ratio) was added to each well. The plate was then incubated again for another 24 h to establish infection. At the end of this period, each well was washed three times with PBS, and then 1 mL of DMEM containing different concentrations of OG (3.12–25 μM) was added, and the plate was incubated for 72 h. After the end of the treatment, the coverslips were collected and stained using a rapid panoptic staining kit and fixed. The samples were analyzed, and 300 macrophages were examined using an optical microscope for each coverslip. An infection index was calculated by multiplying the number of infected macrophages by the number of amastigotes per infected macrophage [15]. For graphical representation of the percentage of infected cells, the raw infection rate of the untreated negative control group was normalized to 100%. The values for the octyl gallate (OG)-treated groups were subsequently calculated and expressed as relative percentages against this baseline. As a positive control, Benznidazole was used. At the end of the experiment, the supernatant was stored in liquid nitrogen for subsequent analysis of cytokines and nitric oxide (NO).

A parallel epimastigote recovery experiment was conducted using the same conditions employed to test intramacrophagic amastigote forms. The assay was performed in 96-well plates, and incubated for 72 h in the presence of OG concentrations (3.12–25 μM). Afterwards, the culture medium was carefully replaced with complete DMEM, without the addition of OG. The plates were then maintained under appropriate culture conditions, allowing the amastigote forms remaining in the cells to transform into trypomastigotes and be released into the extracellular medium. After 72 h, 100 μL of the supernatant containing trypomastigotes was centrifuged and resuspended in LIT medium and transferred to a new plate. This new plate was incubated for 120 h at 28 °C in a BOD chamber, ensuring ideal conditions for the transformation of viable trypomastigotes into epimastigotes, with subsequent proliferation. Finally, the quantification of epimastigotes was performed using a Neubauer chamber.

### 2.5. Cytotoxicity of OG Against LLC-MK2-CCL-7, VERO CCL-81 and RAW 264.7 Macrophages

In 96-well plates, 1 × 10^5^ cells each of LLC-MK2-CCL7, VERO CCL-81, or RAW 264.7 macrophage cell lines were seeded per well in complete DMEM and incubated for 3 h in an incubator at 37 °C with 5% CO_2_ to allow cell adhesion. After this, three washes were performed with PBS to remove non-adherent cells. The supernatant was removed and replaced with a new medium containing serial concentrations of OG, ranging from 800 to 3.12 μM.

The plate was maintained at 37 °C with 5% CO_2_ for 72 h. At the end of the treatment, 10 μL of MTT (5 mg/mL) was added to each well to obtain a final volume of 100 μL per well for colorimetric evaluation of cell viability. The plate was incubated for another 4 h, and the supernatant was then removed and 100 μL of dimethyl sulfoxide (DMSO) was added to dissolve the formazan crystals. Absorbance was measured in a spectrophotometer at a wavelength of 540 nm [16]. The results were expressed as percentage of viability in terms of mean cytotoxic concentration (CC_50_), with the control group treated with complete DMEM containing 0.5% DMSO, considered as 100% cell viability. A positive control was performed using benznidazole.

### 2.6. TR Enzymatic Activity Assay

#### 2.6.1. Computational Details

The TR protein was prepared using CHIMERA version 1.31 software, where all water molecules, ions and residues that were not part of the macromolecule structure were removed from the structure. The three-dimensional (3D) structure of OG was designed and optimized using GaussView 5 and Gaussian 09w software, respectively. The optimization was performed using the Density Functional Theory (DFT) method with the B3LYP hybrid functional and the STO-3G basis set [17].

#### 2.6.2. Molecular Docking

The 3D structure of the Trypanothione Reductase protein was obtained from the Protein Data Bank (PDB) with the code 4NEW. All molecular docking procedures were performed using the Autodock 4.2 package. Protein and ligand were prepared for molecular interaction simulations with AutoDock Tools. Gasteiger partial charges were calculated after the addition of polar hydrogens, and nonpolar hydrogen atoms of the protein and ligand were subsequently added. A 25 × 25 × 25 Å cubic box was generated in the active site of the protein to delineate the interaction site of the ligand with the protein. Other docking parameters were set to default values, and molecular dockings were performed using AutoDock *Vina*. For a more detailed analysis, the complex with the best binding energy obtained was evaluated using Chimera and BIOVIA Discovery Studio Visualizer software, version 21.1.0. [17].

#### 2.6.3. Inhibitory Activity of TR

The TR enzyme inhibition assay was performed following Lima et al. (2015) [18]. To obtain the enzyme, 1 × 10^7^ of parasites were centrifuged twice at 56× *g* for 10 min. The pellet was resuspended in a solution containing 40 mM HEPES (pH 7.5) and 1 mM EDTA (pH 8), lysed in a Dounce homogenizer, and centrifuged at 112× *g* for 15 min. The supernatant obtained was considered the soluble fraction containing TR. Concentrations (mg/mL) were measured using a µQuant Scanning Microplate Spectrophotometer (Biotek-Instrument Inc., Winooski, VT, USA) at 260 and 280 nm. The sample was stored at −70 °C until the assays were performed. To evaluate the effect on TR activity, 1 mg/mL of this soluble fraction was incubated for 6 min with OG at concentrations equivalent to its IC_50_ values of 1×, 2× and 4× its IC_50_ values. The reaction medium to evaluate enzyme inhibition was composed of: 40 mM HEPES pH 7.5, 1 mM EDTA, 100 μM substrate; and 0.5 mM NADPH. Oxidized trypanothione T(S)_2_ (100 mM) was added followed by incubation for 24 h. TR inhibition was assessed by monitoring NADPH consumption, which was observed by reading the optical density at 340 nm in a spectrophotometer.

### 2.7. Ultrastructural Analysis

To perform the analyses, *T. cruzi* epimastigotes in the logarithmic growth phase (1 × 10^7^) were incubated with 1 × CI_50_ OG and conditioned for 24 h in BOD incubator at 28 °C. They were then centrifuged at 3500 *g* for 10 min and at least three washes were performed with PBS, followed by fixation (2% paraformaldehyde and 2% glutaraldehyde) in sodium cacodylate buffer (0.1 M, pH 7.2) for 3 h at room temperature. Afterwards, the samples were centrifuged again (under the same conditions described above) to remove the buffer, and washed in sodium cacodylate buffer (0.1 M, pH 7.2). The samples were post-fixed in 2% osmium tetroxide and 1.6% potassium ferricyanide in 0.2 M sodium cacodylate buffer, pH 7.2, for 30 min at room temperature, and then washed in distilled water. The samples remained overnight at 4 °C in 0.5% uranyl acetate for in bloc counterstaining. Consequently, the samples were dehydrated in increasing concentrations of acetone (30 to 100%). For analysis in a transmission electron microscope (Jeol JEM1011, Tokyo, Japan) after dehydration, the material was embedded in Spurr resin. To obtain ultrathin sections, an ultramicrotome (Leica EM, UC7, Wetzlar, Germany) was used. The sections were contrasted with uranyl acetate and lead citrate. For scanning electron microscopy (SEM) analysis, the samples were dehydrated, dried to the critical point using CO_2_ (Balzers CPD 030, Wallruf, Germany), and sputter-coated with gold (Leica EM SCD 500, Wetzlar, Germany). Imaging was performed using a Jeol JSM-7001F scanning electron microscope (Jeol, Japan) [19].

### 2.8. Cell Death Profile Assay

Trypomastigotes at a concentration of 1 × 10^7^ were incubated with 1×, 2× and 4× the IC_50_ of OG for 24 h at 37 °C. After incubation, the parasites were washed with PBS and labeled with Annexin V and propidium iodide (PI) using the FITC-Annexin V with PI apoptosis detection kit, according to the manufacturer’s instructions. Data acquisition was performed using a BD FACSCanto^®^ II flow cytometer (BD Biosciences, San Jose, CA, USA). A total of 30,000 events were obtained and the results were further analyzed using FlowJo 10.6.2 software (Tree Star, Ashland, OR, USA). Double-negative cells were considered intact, while double-positive cells were considered in late apoptosis/necrotic state. Cells labeled with Annexin V alone were presumably considered to be in early apoptosis, whereas cells labeled with PI alone were considered to be necrotic parasites [15]. Staurosporine (1 µM) was used as a positive control.

### 2.9. Cytokine Production Assay

The supernatant saved from the RAW 264.7 macrophage infection assay was used to evaluate cytokine production. To perform the cytokine analysis, optEIATM ELISA kits (Pharmingen, San Diego, CA, USA) were used following the manufacturer protocol to examine the release of the cytokines (TNF-α, IL-6, IL-10, and IL-12). Recombinant cytokines were used for the standard curve. Absorbance was read in a spectrophotometer with a wavelength of 450 nm [16]. LPS (1 μg/mL) and IFN-γ (1000 U/mL) were used as a positive control.

### 2.10. Reactive Oxygen Species (ROS) Induction and Nitric Oxide (NO) Production Assay

Evaluation of ROS levels in *T. cruzi* infected RAW 264.7 macrophages treated with OG was performed using the H_2_DCFDA assay. For this assay, 100 µL of the supernatant saved from the infection assay was used in a 96-well plate, after which 10 μL of H_2_DCFDA was added to each well to obtain a final concentration of 20 μM. After incubation at 37 °C for 30 min in the dark to avoid photodegradation of the dye, a spectrofluorometer (FLx800) BioTek Instruments (Winooski, VT, USA) was used to read the fluorescence intensity using 485 nm excitation and 528 nm emission. This allowed indirect quantification of macrophage ROS production under the experimental conditions evaluated. As a positive control, lipopolysaccharide (LPS) and interferon-gamma (IFN-γ) were used to stimulate ROS production.

Measurement of NO production (from the macrophage infection supernatant) was performed using Griess reagent to determine nitrite production. Approximately 100 µL of infection supernatant was added to a 96-well plate. In parallel, serial concentrations of NaNO_2_ in DMEM were also added to the plate for interpolation of the standard curve. Griess reagent (1% Sulfanilamide in 10% (*v*/*v*) H_3_PO_4_ in Milli-Q^®^ water and in equal parts 0.1% naphthylenediamine in Milli-Q^®^ water) was then added and incubated for 10 min at room temperature. At the end of the incubation, absorbance was read in a plate reader at 540 nm [20]. LPS (1 μg/mL) and IFN-γ (1000 U/mL) were used as a positive control.

### 2.11. Statistical Analysis

Statistical analyses were performed using GraphPad Prism software (version 8.0; GraphPad Software, San Diego, CA, USA). Data are expressed as mean ± standard deviation (SD) of at least three independent experiments conducted in triplicate.

For the determination of mean inhibitory concentration (IC_50_), mean effective concentration (EC_50_), and mean cytotoxicity concentration (CC_50_) values, non-linear regression analysis was applied directly to the raw, non-normalized absorbance data obtained from the colorimetric assays. A four-parameter logistic curve model (with Hill slope adjustment) was fitted to the complete dataset, utilizing upper and lower constraints (0% and 100% metabolic inhibition plateaus) to ensure mathematically robust estimations of the inflection points. Percentage growth inhibition rates presented in graphical representations were calculated subsequently, solely for relative normalization and visualization purposes against the untreated negative controls.

Differences between experimental groups were evaluated using One-Way Analysis of Variance (ANOVA), followed by Tukey’s post hoc tests for multiple comparisons. Values of *p* < 0.05 were considered statistically significant.

## 3. Results

### 3.1. Anti-Trypanosoma Activity of OG Against T. cruzi Forms

**Figure 2:**
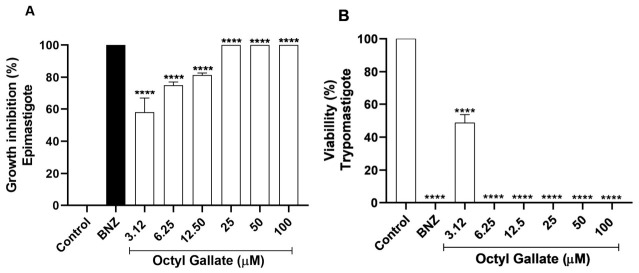
Octyl gallate (OG) activity on epimastigote (**A**) and trypomastigote (**B**) forms of *T. cruzi.* Epimastigote and/or trypomastigote forms (1 × 10^7^) were incubated at 28 °C for 72 h with different concentrations of OG. Growth inhibition was assessed by the MTT test. The results represent the mean ± standard error of three independent experiments performed in triplicate. (****) *p* < 0.0001 vs. the negative control. BZN—benznidazole at a concentration of 200 µM.

The activity of OG against epimastigote and trypomastigote forms are presented in Figure 2. OG promoted a significant inhibition of parasite growth at all concentrations tested. At concentrations of 100 µM, 50 µM, and 25 µM, the compound completely inhibited the growth of epimastigote forms, corresponding to 100% inhibition. Growth inhibition rates were respectively 81.4%, 75%, and 58.1% at concentrations of 12.5 µM, 6.25 µM, and 3.12 µM (Figure 2A), resulting in an IC_50_ of 5.92 µM. Benznidazole completely inhibited (100%) these forms, presenting an IC_50_ of 111.81 ± 6.22 µM (Table 1). Treatment with OG at concentrations of 25, 50, and 100 µM resulted in complete loss of trypomastigote viability, corresponding to 0% viability compared with the untreated control group. The compound promoted a viability rate of 48.75% at the concentration of 3.12 µM, and completely reduced viability (0%) at concentrations of 25 μM, 12.5 μM, and 6.12 μM (Figure 2B). The EC_50_ was determined to be 3.20 ± 0.14 μM (Table 1). Benznidazole inhibited the trypomastigote forms by 100%, obtaining an EC_50_ of 21.11 ± 3.6 μM.

### 3.2. Activity of OG Against Intracellular Amastigotes of T. cruzi

**Figure 3:**
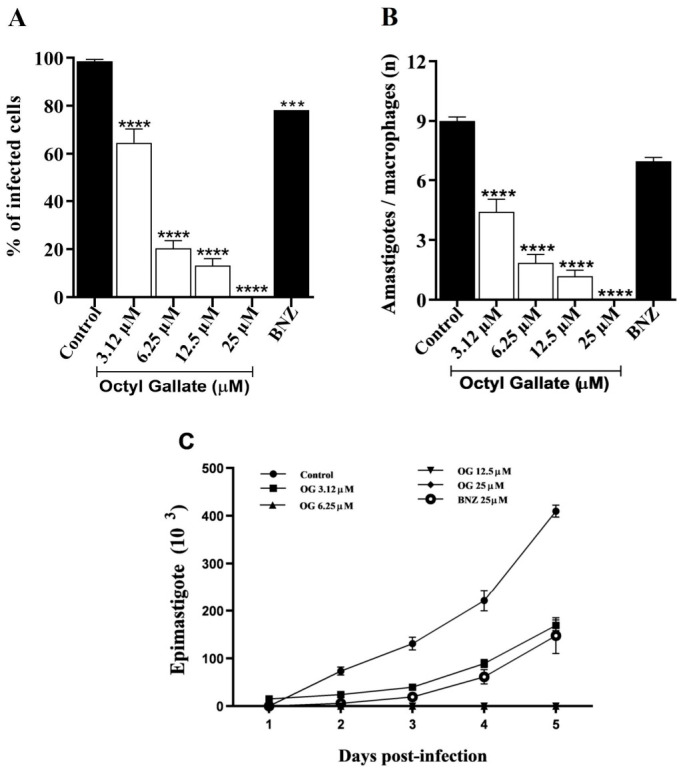
In vitro efficacy of octyl gallate (OG) on infection of RAW 264.7 macrophages by *T. cruzi*. RAW 264.7 macrophages (1 × 10^5^) were infected with trypomastigote forms in a ratio of 10:1 macrophages and treated with OG incubated at 37 °C and 5% CO_2_ for 72 h. (**A**) Percentage of infection, (**B**) number of amastigotes per macrophage, and (**C**) recovery assay. Results represent mean ± standard error of three independent experiments performed in triplicate. (***) *p* < 0.001 vs. the negative control; (****) *p* < 0.0001 vs. the negative control. BZN—benznidazole at a concentration of 25 μM.

Regarding the anti-trypanosomal activity against intracellular forms, RAW 264.7 macrophages were infected with *T. cruzi* trypomastigotes at a multiplicity of infection (MOI) of 10:1 (parasites:macrophage). The effects of OG on the percentage of infected cells and the number of amastigotes per macrophage are shown in Figure 3A,B, respectively. For the percentage of infected macrophages, it was observed that OG was able to reduce the infection rate at all tested concentrations, with reductions of 35.5%, 79.8%, and 87% at concentrations of 3.12 μM, and 6.25 μM, and 12.5 μM, respectively, and a 100% reduction in the infection rate at a concentration of 25 μM, compared to the negative control (Figure 3A).

As for the second criterion, regarding the number of amastigotes per infected macrophage, treatment resulted in a significant reduction in the number of internalized amastigotes by 51.2%, 80%, and 87.8% at concentrations of 3.12 μM, and 6.25 μM, and 12.5 μM, respectively. At the concentration of 25 μM, a reduction of 100% was observed compared to the negative control (Figure 3B).

In order to investigate whether OG exerts trypanocidal or trypanostatic action, an epimastigote recovery assay was performed. After treatment with OG, it was observed that epimastigote forms were unable to develop (even after extended incubation) at the highest concentrations of 6.25, 12.5, and 25 μM (Figure 3C) when compared to the negative control and BZN.

### 3.3. Cytotoxicity of OG on LLC-MK2-CCL-7 and VERO CCL-81 Cells

**Figure 4:**
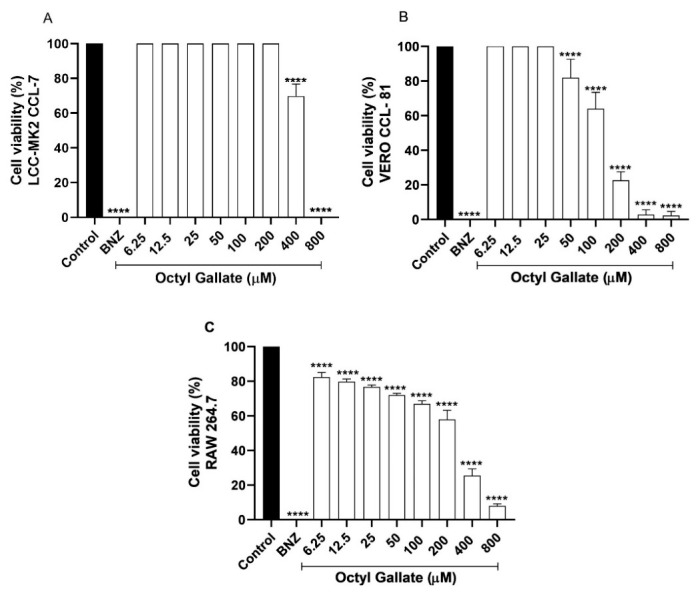
Cytotoxic effects of octyl gallate (OG) on LLC MK2 CCL-7 (**A**), VERO CCL-81 cells (**B**) and RAW 264.7 macrophages (**C**). Cells (5 × 10^5)^ were incubated at 37 °C and 5% CO_2_ for 72 h with different concentrations of OG. Cytotoxicity was evaluated by the MTT assay. Results represent mean ± standard error of three independent experiments performed in triplicate. (****) *p* < 0.0001 vs. negative control. BZN—benznidazole at a concentration of 800 μM.

As shown in Figure 4, the cytotoxicity of OG on mammalian cells was evaluated in LLC-MK2 cell lines, VERO CCL-81 cells, and RAW 264.7 macrophages. It was observed that at concentrations of 12.5 μM, 25 μM, 50 μM, 100 μM, and 200 μM, the LLC-MK2 cells maintained 100% cell viability when compared to the control. However, at the concentration of 400 μM, there was a significant reduction in cell viability, which dropped to 69.77%. At the highest concentration tested (800 µM), no viable cells were observed, indicating a drastic reduction in viability from 100% (Figure 4A), and resulting in a CC_50_ of 417.20 ± 9.27 μM (Table 1).

For the VERO CCL-81 cell line, cell viability was 100% at the concentrations of 12.5 μM and 25 μM. The substance was able to significantly decrease cell viability at concentrations of 50 μM, 100 μM, 200 μM, 400 μM, and 800 μM, yielding respective values of 18.05%, 36.02%, 77.95%, 97.25%, and 97.69%, (Figure 4B), and obtained a CC_50_ value of 116.50 ± 7.31 μM.

In the RAW 264.7 macrophage lineage, it was observed that there was a drastic decrease in cell viability at the highest concentrations tested, with a significant reduction of 74.6% and 92.0% in cell survival at concentrations of 400 µM and 800 µM, respectively (Figure 4C). Consequently, the compound obtained a CC_50_ value of 349.8 ± 5.31 µM. Despite this, OG was markedly more selective for the parasite compared to the reference drugs used in the treatment of the disease (Table 1).

### 3.4. Cytokine Dosage

**Figure 5:**
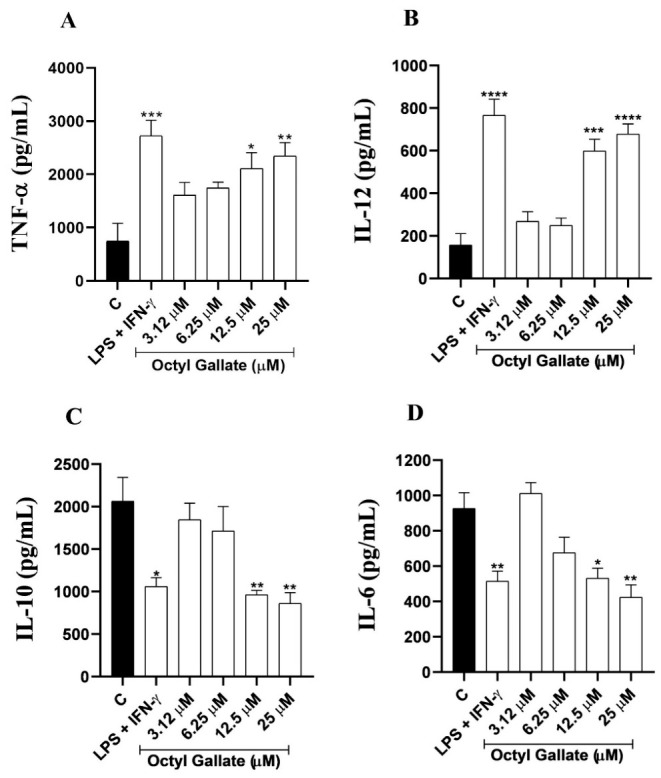
Evaluation of cytokine production in infected macrophages. Levels of cytokines TNF-α (**A**), IL-12 (**B**), IL-10 (**C**), and IL-6 (**D**) produced by uninfected and *Trypanosoma cruzi* trypomastigote-infected macrophages treated with octyl gallate (OG) incubated at 5% CO_2_ at 37 °C for 72 h. Results represent mean ± standard error of three independent experiments performed in triplicate. (*) *p* < 0.05 vs. control; (**) *p* < 0.01 vs. control; (***) *p* < 0.001 vs. control; (****) *p* < 0.0001 vs. control. (C)—control; LPS—*Escherichia coli* lipopolysaccharide (1 μg/mL); IFN-γ—interferon gamma (1000 U/mL).

As illustrated in Figure 5, RAW 264.7 macrophages were first infected with *T. cruzi* to induce cytokine production under inflammatory conditions and subsequently treated with varying concentrations of OG. Treatment with OG at the highest concentrations tested (12.5 and 25 µM) elicited a significant increase in TNF-α and IL-12 production (Figure 5A,B), cytokines typically associated with antiparasitic and pro-inflammatory responses. In contrast, significant reductions in IL-10 (Figure 5C) and IL-6 levels (Figure 5D) were observed at the same concentrations. Together, these findings indicate that OG modulates the cytokine profile of *T. cruzi*-infected macrophages toward a more protective inflammatory response, which may contribute to enhanced parasite control.

### 3.5. Measurement of Reactive Oxygen Species (ROS)

**Figure 6:**
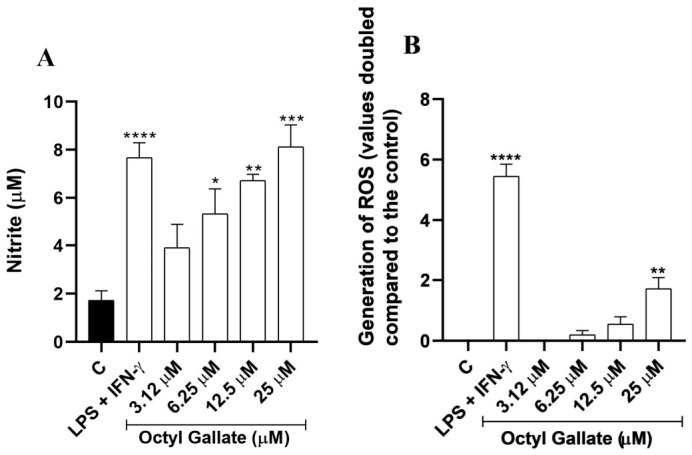
Octyl gallate (OG)-induced changes in cellular mechanisms of RAW 264.7 macrophages. NO (**A**) and ROS (**B**) levels were measured in macrophages treated with OG for 72 h at 37 °C and 5% CO_2_. Results represent the mean ± standard error of three independent experiments performed in triplicate. (*) *p* < 0.05 vs. control; (**) *p* < 0.01 vs. control; (***) *p* < 0.001 vs. control; (****) *p* < 0.0001 vs. control. C—control, LPS—*Escherichia coli* lipopolysaccharide (1 μg/mL), IFN-γ—interferon gamma (1000 U/mL).

The ROS and NO assay results are presented in Figure 6. It An increase in the levels of NO produced by macrophages infected and treated with OG was observed at the three highest concentrations (6.25 μM, 12.5 μM and 25 μM) compared to the negative control (Figure 6A). In relation to the ROS assay, a significant increase was observed only at the highest concentration tested (25 μM) (Figure 6B).

### 3.6. Evaluation of the Effect of OG on TR Activity

#### 3.6.1. Molecular Anchoring

**Figure 7:**
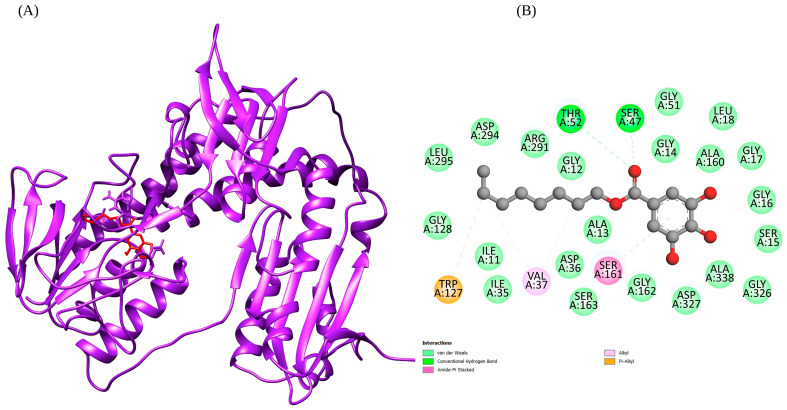
3D molecular docking of the ligand–protein complex with 4NEW. 3D molecular docking of the ligand–protein complex with 4NEW (Chain A color: purple) and octyl gallate (OG) (color: red) illustrating the active binding site (**A**) with the respective interactions (**B**).

The molecular docking results for OG with the trypanothione reductase protein are presented in Table 2. The binding energy parameters were obtained from the OG and 4NEW interaction. An affinity with a binding energy of −7.5 kcal·mol^−1^ was observed (Table 2; Figure 7). Interactions were identified with the amino acids Leu295, Asp294, Arg291, Thr52, Gly12, Ser47, Gly14, Gly51, Ala160, Leu18, Gly17, Gly16, Ser15, Ala338, Gly326, Asp327, Gly162, Ser163, Ser161, Ala13, Asp36, Val37, Ile11, Ile35, Trp127 and Gly128 of the active site of the protein complexed with OG.

#### 3.6.2. Inhibitory Activity of the TR-Rich Fraction

**Figure 8:**
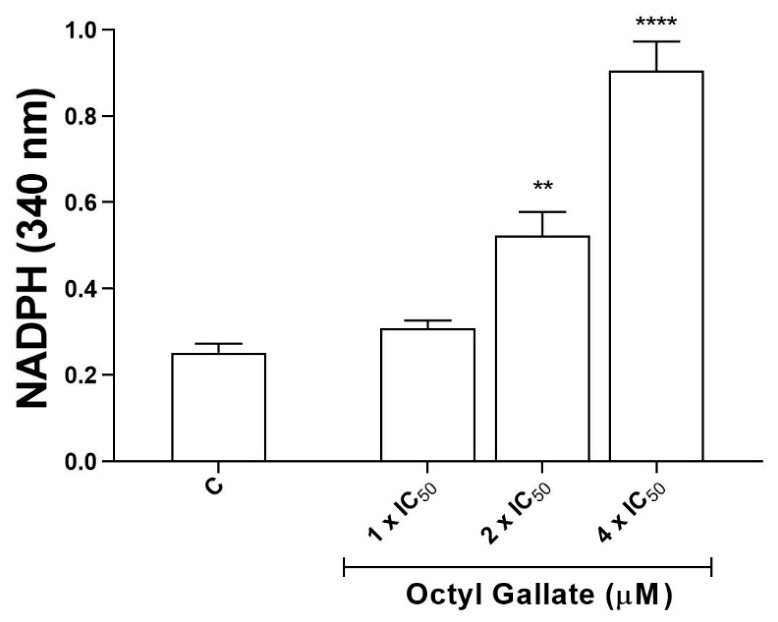
Activity assay of the trypanothione reductase enzyme fraction. Effects of OG on soluble fractions of trypomastigotes. Data represent the mean ± standard error of three independent experiments performed in triplicate, considering the control group containing only the trypanothione disulfide substrate (T(S)_2_) without drugs (**) *p* < 0.01 vs. control; (****) *p* < 0.0001 vs. control.

The results for the inhibition of the TR-rich fraction by OG were evaluated by measuring NADPH consumption, an essential cofactor for the activity of this enzyme. In Figure 8, we can observe a significant increase in the remaining NADPH cofactor at concentrations of 2× and 4× the IC_50_ value in relation to the control. Since reduced NADPH consumption reflects lower enzymatic activity, these results suggest that OG interferes with TR activity.

### 3.7. Ultrastructural Evaluation

**Figure 9:**
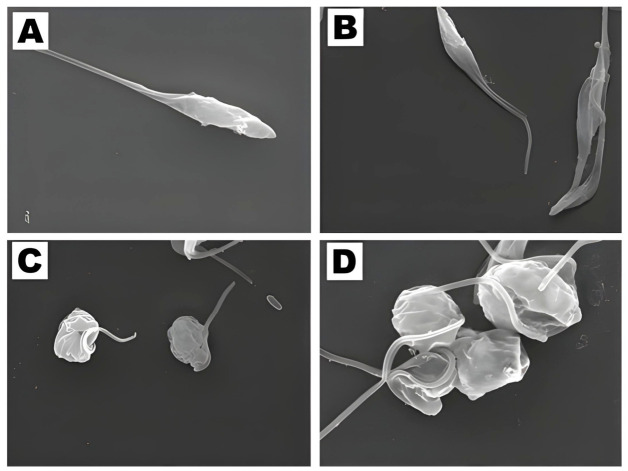
Scanning Electron Microscopy (SEM) of epimastigotes treated with octyl gallate (OG). Epimastigotes were plated in LIT medium, treated with 1 × CI_50_ of OG for 24 h and analyzed by SEM. (**A**,**B**)—negative control; (**C**,**D**)—epimastigotes treated with the compound. Scale bars: 1 µM (**A**–**D**).

In order to elucidate and investigate the changes caused in epimastigote forms treated with OG, SEM and TEM analyses were performed. Figure 9C,D demonstrate the analyses of epimastigote forms of *T. cruzi* treated with OG, evaluated by SEM. Significant changes were observed after treatment; the parasites exhibited wrinkling and shortening of the cell body length, adopting an oval morphology, and an irregular cell surface with possible formation of pores, evidencing a twisting of the body and flagellum (Figure 9C,D). In contrast, the negative control (Figure 9A,B) revealed normal morphology, a regular cell surface, presence of free flagellum, and an elongated and fusiform body.

**Figure 10:**
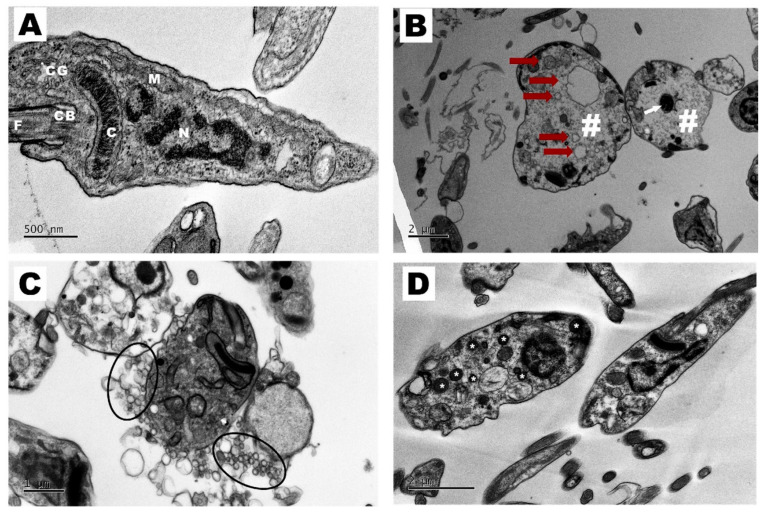
Ultrastructural analysis of epimastigote forms treated with octyl gallate (OG) by Transmission Electron Microscopy (TEM). The epimastigote forms were plated in LIT medium, treated with 1 × IC_50_ of OG for 24 h and analyzed by TEM. (**A**)—negative control; (**B**–**D**)—epimastigotes treated with the compound. (N) Nucleus, (M) mitochondria, (CG) Golgi complex, (CB) basal body, (C) kinetoplast, and (F) flagellum. Red arrow indicates increased vacuoles (**B**); white arrow indicates chromatin compaction and displacement of the nuclear envelope (**B**); # indicates rarefaction of the cytoplasm (**B**); circles represent extravasation of cellular contents (**C**); * represents the appearance of lipid bodies (**D**). Scale bars: 1–500 nm; (**B**,**D**)—2 µM; (**C**)—1 µM.

In the TEM analysis, the negative control (untreated parasites) presented a rounded nucleus and cellular organization, regular kinetoplast morphology, and an intact basal body and flagellum (Figure 10A). The parasites treated with OG presented cellular disorganization, evidencing an increase in the number of cytoplasmic vacuoles, extravasation of cytoplasmic content, and a rarefied cytoplasm (Figure 10B,C), the presence of electron-dense structures suggestive of lipid bodies (Figure 10D), detachment of the nuclear membrane, increased chromatin condensation, and nuclear fragmentation (Figure 10B).

### 3.8. Cell Death Profile Assessment

**Figure 11:**
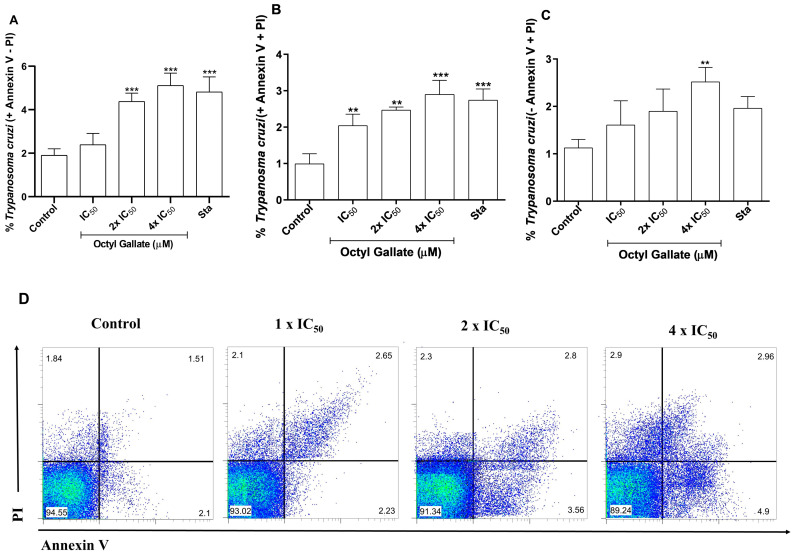
Evaluation of the death profile of *Trypanosoma cruzi* trypomastigote forms treated with Octyl gallate (OG) by flow cytometry. Trypomastigote forms were incubated for 24 h with OG at concentrations of 1×, 2×, and 4× the IC_50_. Double labeling with Annexin V/IP was performed and evaluated by flow cytometry. (**A**) annexin V-FITC+/PI- staining patterns; (**B**) annexin V-FITC+/PI+ staining patterns; (**C**) annexin V-FITC-/PI+ staining patterns; and (**D**) representative dot plots showing staining of *T. cruzi* trypomastigotes. Data represent the mean ± standard error. Comparison between groups was performed by One-way ANOVA followed by Tukey’s post-test, with (**) *p* < 0.01 vs. control; (***) *p* < 0.001 vs. control. Sta—Staurosporine at 1 µM.

To better analyze parasite death, we performed flow cytometric measurements of early and late apoptotic parameters, and necrosis after 24 h of exposure to OG. The parasites were double labeled with Annexin V and PI. In the trypomastigote assay, there was a significant increase in the percentage of cells labeled with Annexin V at 2× and 4× the IC_50_ concentrations, evidencing an early apoptosis process (Figure 11A). Double labeling with Annexin V and PI was also observed for all IC_50_ concentrations tested, thus inferring late apoptosis (Figure 11B). In Figure 11C, we can observe that there was labeling with PI only at the 4× IC_50_ concentration, demonstrating a possible secondary cell death by necrosis.

## 4. Discussion

In the present study, the anti-trypanosome activity of OG was evaluated. First, the biological capacity of the substance to inhibit the epimastigote forms of the parasite was analyzed. Epimastigotes are replicative forms present only in the digestive tract of the insect vector. However, most cell biology studies and in vitro tests for new chemotherapeutics are performed with epimastigotes, since they develop in axenic culture media and are easily maintained in the laboratory [21]. Epimastigote forms are convenient for most cell biology studies, since many metabolic pathways are similar between the different forms of the parasite, and for drug testing, facilitating initial toxicity screening [21]. The test against this evolutionary form was performed as an initial screening, and it was observed that OG was able to significantly inhibit parasite growth at low concentrations. Andréo and collaborators in their studies evaluated 13 gallate esters, six of which (containing alkyl chains ranging from 6 to 14 carbons) demonstrated activity against the epimastigote forms of *T. cruzi.* [22]. These results corroborate the studies of Fujita and collaborators [23], who reported that the trypanocidal activity becomes more potent as the carbon chain length increases, thereby confirming the relationship between biological activity and chain length.

The trypomastigote form has great clinical relevance, as it is the evolutionary form which infects humans and circulates free in the host bloodstream [24]. The ability of OG to inhibit this evolutionary form was therefore evaluated. The study demonstrated even more effective trypanocidal activity against these forms, presenting a lower EC_50_ than other compounds considered potential trypanocidal agents. Amisigo et al. [25] tested gallic acid, a precursor of OG, against *T. brucei*, observing significant inhibition with an IC_50_ value of 14.2 μM. Ferreira and collaborators [26] tested the trypanocidal effect of certain phenolic compounds, including methyl and propyl gallates (which presented IC_50_ of 10.4 μM and 15.7 μM, respectively), and observed a positive correlation between inhibition of the respiratory chain and reduction in the proliferation of blood trypomastigote forms of *T. brucei.* The authors suggested that some phenolic compounds can interrupt the electron transport mediated by ubiquinone/ubiquinol, mimicking coenzyme-Q, a mechanism of action proposed by Grady and collaborators [27] for *T. brucei.* This suggests a possible mechanism of action involved in the activity of OG.

Another possible explanation for this result is that certain gallates may act as surfactants, interfering with the function of the parasite’s membrane proteins [28]. According to Souza et al. [29], the esters could be causing the inhibition of ergosterol biosynthesis, which is essential for the survival of the parasite. The results of Abe et al. [30] demonstrated that certain gallates have the ability to inhibit the enzyme squalene epoxidase, which plays a crucial role in the process of ergosterol biosynthesis. Further studies by Lazardi et al. [31] and Mosmann [32] indicate that ergosterol inhibitors, especially those acting on the enzyme squalene epoxidase, can cause changes in the parasite’s kinetoplast-mitochondria complex.

The viability (or functional status) of cells is generally assessed by techniques used to determine the cytotoxicity of a chemical agent. Quantitative tests based on colorimetric changes are extremely versatile, and were developed for use with microplate readers, also known as multichannel spectrophotometers [33]. Such tests allow a large number of substances to be measured simultaneously with high precision. They are based on substrates which, after being metabolized by living cells, produce a product with characteristics different than the initial substrate. The process does not occur in dead cells and may require changes in the components of the culture medium [34].

The cytotoxicity assay was evaluated in VERO cells (immortalized fibroblasts from African green monkey kidneys) [35], LLC-MK2 cells (an epithelial cell line obtained from Rhesus monkey kidneys) [36], and RAW 264.7 macrophages. In the present study, GO demonstrated distinct CC_50_ values across the evaluated cell lines, all of which were higher than the IC_50_ and EC_50_ values, implying that the substance is more selective for the parasite than for host cells. Melo and collaborators [37] observed that isopropyl gallate, a compound belonging to the same class as GO, showed low toxicity in RAW 264.7 cells, obtaining a CC_50_ of 1260 μM. Studies conducted by Alves and collaborators [38] demonstrated that the OG precursor, gallic acid, showed low toxicity in macrophages, with CC_50_ of 744.18 µM, thereby ensuring greater selectivity for the parasite. Together, these findings underscore the favorable safety profile of OG in vitro, establishing it as a promising and highly selective prototype for further anti-trypanosomal drug development.

In comparison with the reference drug benznidazole, OG exhibited lower potency against some parasite forms, as evidenced by the lower IC50 values reported for benznidazole in several in vitro studies. However, OG demonstrated markedly higher selectivity toward *T. cruzi*, presenting selectivity indices approximately ten-fold higher than those observed for the reference drug. Benznidazole remains the first-line treatment for Chagas disease despite important limitations, including adverse effects, variable susceptibility among parasite strains, and reduced efficacy during the chronic phase of infection [39].

Once the concentrations capable of inhibiting parasite growth in a safe manner for cells were found, experiments were performed with intramacrophagic amastigotes, a model that most closely resembles in vivo efficacy. As a result of treatment with OG, a significant reduction in the number of infected macrophages and a reduction in the number of intramacrophagic amastigotes were observed. Panaro and collaborators [40] reported in their studies that compounds of the gallate class are capable of reaching parasitophorous vacuoles at adequate levels, maintaining their antiparasitic effects and eliminating the parasite, and stimulating potential effects of the compound on cell, activating microbicidal pathways, and acting on the parasite without altering cell viability.

The host immune response against *T. cruzi* is crucial for manifestation of the various clinical presentations of the disease [15]. Throughout the different phases of infection, a specific immune response occurs, characterized by a repertoire of cells, cytokines and other substances that play a dual role. For while they contribute to the reduction in the parasite load and the protection of the organism, they may also be involved in the pathogenesis of the disease [41].

The inflammatory process begins with infection, and the elimination of intracellular pathogens requires the participation of immune system cells and cytokines characteristic of a cell-type response mediated by T helper 1 lymphocytes (Th1) [36]. Correlating with the immunomodulatory response, OG was able to stimulate the production of TNF-α and IL-12 in infected and treated macrophages, which corroborates the hypothesis that the compound is able to stimulate activation of the Th1 pathway, helping to fight the parasite. Trott et al. [42], using murine macrophages as host cells, observed that the presence of recombinant TNF-α prevented the replication of *T. cruzi.* Silva and collaborators [43], investigating the role of TNF-α in resistance to infection by *T. cruzi* in a line of resistant mice, observed that the use of a TNF-α monoclonal antibody led to the susceptibility of mice to the parasites.

On the other hand, IL-10 is associated with susceptibility to infection by inhibiting macrophage activation. These findings suggest that IL-10 may be a potent inhibitor of IFN-γ and TNF-α production during *T. cruzi* infection [41]. It is also known that IL-6 is associated with a more severe immune response, related to the development of the chronic cardiac form of the disease [36]. As to the association with Th2 profile cytokines, such as IL-10 and IL-6, it was observed that OG (at the highest concentrations tested) exhibited a remarkable capacity to significantly suppress the production of these cytokines, compared to the untreated control group. This inhibitory effect on the synthesis of the cytokines in question corroborates the promotion of parasite control, since regulation of these immunological mediators results in a response against the parasite, with consequent benefits for the host [44].

In addition to the immunomodulatory response, other cellular mechanisms, such as the production of ROS and NO play a fundamental role in the response against invading organisms. As phagocytes are exposed to IFN-γ, the classic activation and induction of microbicidal mechanisms may occur, with the participation of the enzyme inducible nitric oxide synthase (iNOS), an enzyme that synthesizes NO, a mediator of inflammation, which together with other ROS and reactive nitrogen species, controls the parasite load [29]. In the present study, it was observed that OG increases the production of ROS and NO by macrophages infected with *T. cruzi*, at the same concentrations at which it decreases the parasite load of macrophages. This suggests the participation of these mechanisms in its trypanocidal activity.

Parasites have developed many defense mechanisms that allow them to survive in completely hostile intracellular environments. Some of these mechanisms depend on an antioxidant defense enzyme system composed of a thiol-TR [45]. TR is an essential enzyme for the survival of trypanosomatids, maintaining trypanothione in its reduced form. This reduces the levels of oxygen free radicals (ROS) and contributes to maintaining a reducing intracellular environment. The enzyme is the main defense mechanism of trypanosomatids against oxidative stress and is absent in mammalian cells [46].

Docking techniques were used to evaluate the interaction of TR with OG. The integration of computational and experimental strategies allows identification and development of new promising compounds, and this methodology reduces research time, in addition to minimizing the financial costs involved [46]. Among these strategies, molecular docking stands out in modern drug design due to its ability to predict, with considerable accuracy, the conformations and orientations of small molecules (ligands) in the binding sites of macromolecular targets, such as proteins, known as receptors. The analysis of the interaction between the ligand and the receptor, especially in the context of enzymatic reactions, is considered a crucial step to identify possible inhibitors and, thus, develop new therapeutic approaches for many important pathologies [47].

In the present work, the interaction between OG and TR was investigated, and a good and strong docking connection between ligand and receptor was observed. According to Trott et al. [42], binding energy values lower than −5 kcal/Mol indicate good ligand/receptor interaction and values lower than −7 kcal/Mol guarantee greater stability of the bond. OG presented higher values than those found in the literature, thus demonstrating a strong and stable bond. The presence of hydrogen bonds reinforces the stability and specificity for the active site of TR. These findings corroborate the findings of Mehwish et al. [48], who tested in silico the relationship of TR with the OG precursor, gallic acid, and observed a great interaction with the ligands. A general molecular docking investigation revealed that the ligands present good interactions with important catalytic residues of the target protein.

To validate the in silico findings experimentally, an in vitro inhibition test of the soluble fraction of TR was performed. It was observed (through the measurement of NADH) that OG was able to significantly inhibit the production of TR in trypomastigote forms of *T. cruzi* at the highest concentrations tested. Mehwhis and collaborators [48] reported in their studies that gallic acid was able to inhibit 93% of the enzymatic activity of TR in *Leishmania braziliensis* at a concentration of 100 µM, thus evidencing its activity. These results demonstrate the effectiveness of OG in inhibiting TR, a crucial enzyme for the development of the parasite, and triggering a decrease in parasitic proliferation.

Various strategies can be employed to investigate the activities of new molecules and identify specific targets when developing drugs for the treatment of CD. Among these strategies, microscopy stands out for its ability to assist detailed analyses of the ultrastructural modifications induced by experimental compounds. Microscopy allows identification of relevant targets in organelles, metabolic pathways and structural damage to the membrane, and additionally, can offer important input towards elucidation of mechanisms of action of substances known to be active against *T. cruzi* [49].

Epimastigote forms of *T. cruzi* were analyzed by SEM and TEM to observe structural changes potentially caused by OG. Analyses of the extracellular morphology of the parasite were performed using SEM, evidencing significant structural changes, such as the wrinkling and shortening of the cell body, and processes giving an oval shape to the body, in addition to the possible formation of pores in the membrane. According to study by Ali et al. [50], the loss of membrane integrity is suggestive of apoptosis-induced cell death. TEM assays were used to observe intracellular damage caused by OG, and the studied compound was shown to cause cellular disorganization, an increase in cytoplasmic vacuoles, and leakage of the content, confirming the presence of pores in the membrane. Nuclear damage was observed, with subsequent displacement of the membrane, fragmentation, and condensation of the nucleus. Vannier-Santos and collaborators [49] reported that substances capable of causing such changes in treated parasites can be associated with the apoptotic death pathway, since these changes can be considered indicators of programmed cell death.

For cell death profile analysis, a double-labeling assay with Annexin V/IP was used. Annexin V is widely used to label cells undergoing apoptosis. During apoptosis, phosphatidylserine translocates from the inner to the outer face of the plasma membrane, allowing binding with the marker [49]. However, the propidium iodide probe cannot cross intact plasma membranes. It penetrates only cells with damaged membranes, binding to DNA to label dead or nonviable cells [51]. Thus, double-labeling with Annexin V/IP allowed the differentiation of apoptotic from necrotic parasite populations.

Regarding the direct killing mechanisms, double-labeled trypomastigotes subjected to OG treatment demonstrated a cell death profile likely triggered through an initial apoptotic pathway, progressing to secondary necrosis at higher concentrations. This corroborates the findings of Cortes et al. [52], who reported that gallic acid was able to induce cell death by apoptosis in *T. cruzi* strain Y trypomastigotes at low concentrations. According to Machado et al. [53], the components present in phyto-pharmaceuticals and/or semi-synthetic products can induce changes in parasite metabolism which lead to cell death through the autophagy process, degrading the parasite’s structures. The results obtained in this study allow us to infer that OG has the potential to serve towards treatment of CD, encouraging further studies in experimental in vivo models.

## 5. Conclusions

Based on the results obtained, it can be inferred that OG demonstrated high selectivity and efficacy in inhibiting the different evolutionary forms of *T. cruzi*, achieving a performance superior to that of the reference drug benznidazole. Its trypanocidal activity is mechanistically associated with inhibition of the TR enzyme, deregulating the redox balance of trypanosomatids, and with immunomodulatory activity, activating microbicidal functions and stimulating a protective immune response in host cells. Our results suggest that OG presents trypanocidal potential worthy of further research to develop new treatments for CD. However, additional studies are needed to validate its efficacy in vivo and to better understand its mechanisms of action, as well as to investigate its bioavailability and safety in experimental models.

## Figures and Tables

**Table 1:** Anti-trypanosomal activity, cytotoxic effect and selectivity index (SI) values calculated for octyl gallate (OG) and benznidazole (BZN).

Compounds	MK2	VERO	RAW 264.7	Epimastigote				Trypomastigote				Intracellular Amastigotes			
	CC_50_ µM	CC_50_ µM	CC_50_ µM	IC_50_ µM	SI_MK2_	SI_VERO_	SI_RAW_	EC_50_ µM	SI_MK2_	SI_VERO_	SI_RAW_	EC_50_ µM	SI_MK2_	SI_VERO_	SI_RAW_
**OG**	417.20 ± 9.27	116.50 ± 7.31	349.8 ± 5.31	5.92 ± 0.47	70.47	19.67	59.08	3.20 ± 0.14	130.37	36.40	109.3	4.07 ± 0.72	102.5	28.6	85.9
**BZN**	614.81 ± 15.9	147.37 ± 9.63	79 ± 7.63	111.81 ± 6.22	5.49	1.32	0.71	21.11 ± 3.6	2.91	6.98	3.76	24 ± 0.27	25.6	6.14	3.29

IS = CC_50/_CI_50_, or CE_50_.

**Table 2:** Molecular affinity parameters for octyl gallate (OG) with the 4NEW protein.

Ligand–Protein Complex	ΔG Bond ^a^ (kcal·mol^−1^)	Ligand–Protein Residue Interactions ^b^
OG-4NEW	−7.5	Leu295, Asp294, Arg291, Thr52, Gly12, Ser47, Gly14, Gly51, Ala160, Leu18, Gly17, Gly16, Ser15, Ala338, Gly326, Asp327, Gly162, Ser163, Ser161, Ala13, Asp36, Val37, Ile11, Ile35, Trp127, and Gly128

^a^ power bond in the best conformation; ^b^ Obtained with BIOVIA Discovery Studio Visualizer.

## Data Availability

Dataset available on request from the authors.
